# Cannabidiol Acts at 5-HT_1A_ Receptors in the Human Brain: Relevance for Treating Temporal Lobe Epilepsy

**DOI:** 10.3389/fnbeh.2020.611278

**Published:** 2020-12-15

**Authors:** Christopher Martínez-Aguirre, Francia Carmona-Cruz, Ana Luisa Velasco, Francisco Velasco, Gustavo Aguado-Carrillo, Manola Cuéllar-Herrera, Luisa Rocha

**Affiliations:** ^1^Department of Pharmacobiology, Center for Research and Advanced Studies, Mexico City, Mexico; ^2^Epilepsy Clinic, Hospital General de México Dr. Eduardo Liceaga, Mexico City, Mexico

**Keywords:** serotonin, 5-HT_1A_ receptor, hippocampus, mesial temporal lobe epilepsy, drug-resistant epilepsy, cannabidiol, temporal neocortex

## Abstract

Experimental evidence indicates that cannabidiol (CBD) induces anxiolytic and antiepileptic effects through the activation of 5-HT_1A_ receptors. These receptors are coupled to G_i/o_ proteins and induce inhibitory effects. At present, the interaction of CBD with 5-HT_1A_ receptors in the human brain is unknown. The aim of this study focused on evaluating the interaction between CBD and 5-HT_1A_ receptors in cell membranes obtained from the hippocampus and temporal neocortex of autopsies and patients with drug-resistant mesial temporal lobe epilepsy (DR-MTLE). Cell membranes were isolated from the hippocampus and temporal neocortex of a group of patients with DR-MTLE who were submitted to epilepsy surgery (*n* = 11) and from a group of autopsies (*n* = 11). The [^3^H]-8-OH-DPAT binding assay was used to determine the pharmacological interaction of CBD with 5-HT_1A_ receptors. The [^35^S]-GTPγS assay was used to investigate the CBD-induced activation of G_i/o_ proteins through its action on 5-HT_1A_ receptors.The CBD affinity (p*K*_i_) for 5-HT_1A_ receptors was similar for autopsies and patients with DR-MTLE (hippocampus: 4.29 and 4.47, respectively; temporal neocortex: 4.67 and 4.74, respectively). Concerning the [^35^S]-GTPγS assay, no statistically significant changes were observed for both hippocampal and neocortical tissue (*p* > 0.05) at low CBD concentrations (1 pM to 10 μM). In contrast, at high concentrations (100 μM), CBD reduced the constitutive activity of G_i/o_ proteins of autopsies and DR-MTLE patients (hippocampus: 39.2% and 39.6%, respectively; temporal neocortex: 35.2% and 24.4%, respectively). These changes were partially reversed in the presence of WAY-100635, an antagonist of 5-HT_1A_ receptors, in the autopsy group (hippocampus, 59.8%, *p* < 0.0001; temporal neocortex, 71.5%, *p* < 0.0001) and the group of patients with DR-MTLE (hippocampus, 53.7%, *p* < 0.0001; temporal neocortex, 68.5%, *p* < 0.001). Our results show that CBD interacts with human 5-HT_1A_ receptors of the hippocampus and temporal neocortex. At low concentrations, the effect of CBD upon G_i/o_ protein activation is limited. However, at high concentrations, CBD acts as an inverse agonist of 5-HT_1A_ receptors. This effect could modify neuronal excitation and epileptic seizures in patients with DR-MTLE.

## Introduction

Cannabidiol (CBD), the main non-psychoactive component of *Cannabis* plants (Russo, 2017), has a terpenophenolic structure hydroxylated at carbons 1 and 3 (Jones et al., 1977). This structure gives CBD lipophilic properties, which allow its passage across the blood–brain barrier (Calapai et al., 2020).

CBD induces antiepileptic effects in humans and experimental models (Silvestro et al., 2019). In patients with Dravet- or Lennox-Gastaut syndromes, the administration of CBD reduces the frequency and severity of the seizures (Maa and Figi, 2014; Thiele et al., 2018; Lazaridis et al., 2019). In patients with drug-resistant temporal lobe epilepsy (DR-TLE), the coadministration of CBD with antiseizure drugs reduces the number and intensity of epileptic seizures (Cunha et al., 1980). CBD administration also decreases seizure activity and neuronal hyperexcitability in experimental models of temporal lobe epilepsy (Khan et al., 2018; Patra et al., 2019). Moreover, CBD produces anxiolytic effects in humans and experimental models (Shannon et al., 2019). CBD induces antidepressant effects that are more evident when tissue serotonin levels are high (Sales et al., 2018). These effects are partially explained because CBD interacts with 5-hydroxytryptamine1A (5-HT_1A_) receptors (Magen et al., 2010). In cultured Chinese hamster ovary (CHO) cells expressing 5-HT_1A_ receptors, CBD showed micromolar affinity in displacing [^3^H]-8-OH-DPAT from 5-HT_1A_ receptors, increased [^35^S]-GTPγS binding in this G_i/o_ protein-coupled receptor, and reduced cAMP concentration. According to these results, the authors concluded that CBD behaves as an agonist of 5-HT_1A_ receptors (Russo et al., 2005). These effects were not reproduced when cell membranes obtained from rat brainstem were exposed to CBD. However, this phytocannabinoid enhanced the ability of 8-OH-DPAT, an agonist of 5-HT_1A_ receptors, to stimulate [^35^S]-GTPγS binding. These results suggest that CBD shows an allosteric interaction with 5-HT_1A_ receptors (Rock et al., 2012).

At present, the interaction of CBD with 5-HT_1A_ receptors in the human brain is not known. Indeed, changes of this interaction induced by drug-resistant epilepsy may represent a condition that augments or reduces the efficacy of CBD to control the seizure activity. The aim of this study focused on evaluating the interaction of CBD with 5-HT_1A_ receptors in cell membranes obtained from the hippocampus and temporal neocortex of patients with drug-resistant mesial TLE (DR-MTLE). The results were compared with brain tissue with no neurological disorders obtained from autopsies.

## Materials and Methods

### Patients with DR-MTLE

Patients with DR-MTLE underwent an extensive presurgical evaluation that included video electroencephalogram (EEG) and magnetic resonance imaging (MRI) in the Epilepsy Clinic of the General Hospital from Mexico Dr. Eduardo Liceaga. Four serial EEGs were conducted to determine the presence and location of epileptiform activity. T1–T2-weighted MRI served to identify mesial sclerosis. Patients with cortical dysplasia or neocortical TLE were excluded from the study. After the presurgical evaluation, 11 patients with DR-MTLE (five females and six males) were included in the present study. The scientific and ethics committees from all the institutions involved approved this experimental protocol (authorization number DI/15/403/03/32). Written informed consent was obtained from all participants.

A standard anterior temporal lobectomy ipsilateral to the epileptogenic zone was performed in every patient 48 h after the last seizure occurrence. During the surgical procedure, samples from the hippocampus and temporal neocortex were collected immediately after resection and stored at −70°C (Table 1).

**Table 1:** Clinical data of patients with drug-resistant mesial temporal lobe epilepsy (DR-MTLE).

ID	Gender	Age (years)	Age of seizure onset (years)	Duration of epilepsy (years)	Frequency of seizures (per month)	Side of focus	Precipitating factors	ASD before surgery
HUM-138	F	37	4	33	75	Left	Febrile seizures in childhood	PHT/CBZ/VA/ OXC/CLN
HUM-154	F	9	3	6	7	Right	Febrile seizures in childhood	VA/TOP/CBZ/ PHB/LEV/LAM
HUM-155	M	31	12	19	20	Left	Febrile seizures in childhood	PHT/VA/CBZ/LAM
HUM- 161	M	19	0.16	19	2	Left	Febrile seizures in childhood	PHT/VA/CBZ/TOP
HUM-162	M	56	18	38	30	Right	Temporal-occipital AVM	CBZ/DZP/PHT/ PHB/LEV/VA
HUM-168	M	28	15	13	15	Bilateral	TBI and mother with epilepsy	CBZ/CLN/LEV
HUM-169	M	46	12	34	15	Right	TBI	PHT/CBZ
HUM-173	F	46	8	38	2	Right	Unknown	PHT/OXC
HUM-184	F	28	13	15	16	Bilateral	Unknown	LAM/VA/CBZ/LEV
HUM-192	M	41	32	9	10	Left	Father with epilepsy	LAM/VA/CBZ/LEV
HUM-193	F	22	11	11	4	Right	Febrile seizures in childhood	LAM/VA/LEV/TOP

*ASD, antiseizure drugs; AVM, arteriovenous malformation; CBZ, carbamazepine; CLN, clonazepam; F, female; LAM, lamotrigine; LEV, levetiracetam; M, male; OXC, oxcarbazepine; PHB, phenobarbital; PHT, phenytoin; TBI, traumatic brain injury; TOP, topiramate; VA, valproic acid*.

### Autopsies

Samples from the hippocampus and temporal neocortex were obtained from 11 autopsies (three females and eight males, 37.1 ± 17.8 years of age). Death was not associated with neurological disorders, for which brain tissues were analyzed and considered as controls. Autopsy samples were collected with a postmortem interval (PMI) of 15.91 ± 3.11 h. The samples were frozen immediately after resection and stored at −70°C. Autopsies were performed at the Institute of Forensic Sciences in Mexico City (Table 2).

**Table 2:** Clinical data of autopsies.

ID	Gender	Age (years)	Cause of death	PMI (h)
A2	M	29	Polycontusion	18
A3	M	30	Ballistic trauma	14
A7	M	45	Suffocation	18
A8	M	73	Complications associated with diabetes	15
A10	M	36	Ballistic trauma	12
A11	F	12	Suffocation	14
A12	F	40	Ballistic trauma	20
A14	F	45	Unknown	10
A16	M	57	Myocardial infarction	18
A17	M	25	Penetrating wound in thorax	18
A21	M	16	Suffocation	18

*F, female; h, hours; M, male; PMI, postmortem interval*.

### Radioligand Displacement Assay

To assess the interaction of CBD with 5-HT_1A_ receptors, we conducted radioligand displacement assays and evaluated the ability of CBD to displace a radioactively labeled ligand bound to these receptors. Cell membranes were obtained as previously described, with some modifications (Benyhe et al., 1997). Briefly, brain tissue (~500 mg) was homogenized in ice-cold 50 mM Tris–HCl buffer (pH 7.4). Subsequently, it was centrifuged at 15,000 rpm for 25 min at 4°C. The resulting pellet was resuspended in 50 mM Tris–HCl buffer (pH 7.4) and incubated for 30 min at 35°C. At the end of the incubation, the preparation was centrifuged under the conditions previously indicated. The resultant pellet was resuspended in buffer (50 mM Tris–HCl, 1 mM EGTA, and 5 mM MgCl_2_•6H_2_O, pH 7.4), and protein concentration was determined by the Bradford method (Bradford, 1976).

The radioligand displacement assay was performed in a final reaction volume of 500 μl that contained 250 μg of protein of the membrane suspension and increasing concentrations of CBD (10 nM to 10 mM). The assay was carried out in the presence of [^3^H]-8-OH-DPAT at 0.7 nM. This ligand has a high affinity for 5-HT_1A_ (*K*_i_ = 0.56) and low affinity for other receptors (*K*_i_ ranging from 41.9 to >10,000; Middlemiss and Fozard, 1983; Schlegel and Peroutka, 1986; Brown et al., 1990; Pauwels et al., 1996; Kleven et al., 1997). According to the *K*_i_ of [^3^H]-8-OH-DPAT for the different receptors, we expected that the results obtained represent the interaction of this ligand on 5-HT_1A_ receptors.

The mixture was incubated for 45 min at 35°C. Non-specific binding was determined in the presence of the 5-HT_1A_ receptor antagonist WAY-100635 (10 μM). The reaction was terminated by rapid filtration on a Brandel M-48 multifilter through a Whatman GF/C glass fiber filter followed by three washes with ice-cold buffer (50 mM Tris–HCl, pH 7.4). Radioligand binding was determined as disintegrations per minute (DPM) values (Beckman LS6000SC scintillation counter), which were normalized with respect to the maximum binding. Data were fitted to a non-linear regression to determine the inhibitory concentration 50 (IC_50_) with the model *Y* = Bottom + (Top − Bottom)/(1 + 10^(LogIC50-X) * HillSlope^) using Prism software (GraphPad Software, Inc.). The same equation was used to determine the Hill coefficient, which gives information on the number of interacting sites and possible allosteric interactions (Prinz, 2010). The Cheng–Prusoff equation was used to determine the inhibition constant (*K*_i_) considering the dissociation constant (*K*_d_) of [^3^H]-8-OH-DPAT equal to 0.46 nM (Cheng and Prusoff, 1973; Cusack et al., 1994). Data are expressed as the mean ± standard error of the mean (SEM).

### [^35^S]-GTPγS Binding Assay

5-HT_1A_ receptors are highly expressed G_i/o_ protein-coupled receptors that activate inhibitory pathways. Considering that CBD acts on several G_i/o_ protein-coupled receptors, such as CB_1_, CB_2_, opioid, and 5-HT_1A_, among others (Alves et al., 2020), we initially evaluated if CBD was able to activate the G_i/o_ protein and if a specific antagonist to 5-HT_1A_ receptors was able to prevent such effect. The [^35^S]-GTPγS binding assay was used for this purpose.

Brain tissue was homogenized in buffer (50 mM Tris–HCl, 1 mM EGTA, and 3 mM MgCl_2_•6H_2_O, pH 7.4). The homogenate was centrifuged at 20,000 rpm for 45 min at 4°C. The resulting pellet was resuspended in buffer (50 mM Tris–HCl, 0.2 mM EGTA, 9 mM MgCl_2_•6H_2_O, and 150 mM NaCl, pH 7.4) and centrifuged under the same conditions. The resulting pellet was resuspended in buffer once more, and protein concentration was determined as described above (see “Radioligand Displacement Assay” section).

The [^35^S]-GTPγS binding assay was carried out as previously described (Spetea et al., 1998; Cuellar-Herrera et al., 2014), with minor variations. Briefly, cell membranes (10 μg of protein) were incubated at 30°C for 60 min in a reaction tube that contained Tris–EGTA buffer [Tris–50 mM HCl, 1 mM EGTA, 3 mM MgCl_2_•6H_2_O, 100 mM NaCl, and 0.1% (w/v) albumin; pH 7.4], GDP (100 μM), [^35^S]-GTPγS (100 μM), and increasing concentrations of CBD (1 pM to 100 μM). Total binding was measured in the absence of CBD. Non-specific binding was estimated in the presence of unlabeled GTPγS (100 μM). Data were analyzed as specific binding that resulted from subtracting non-specific binding from total binding. If any effect was obtained, WAY-100635 (100 μM) was included in the assay to determine the participation of 5-HT_1A_ receptors. The reaction was terminated by rapid filtration on a Brandel M-48 multifilter through a Whatman GF/B glass fiber filters followed by three washes with ice-cold buffer (50 mM Tris–HCl and 5 mM MgCl_2_•6H_2_O, pH 7.4). A concentration–effect curve was built with the percentage of activation calculated considering the basal binding (in the absence of stimulation) as zero. Data were analyzed by a two-way ANOVA, taking group and CBD concentration as factors (concentration–effect curve), or a one-way ANOVA (effect of 100 μM in presence or absence of WAY-100635). *p* values < 0.05 were considered statistically significant.

## Results

### CBD Acts on 5-HT_1A_ Receptors in Human Brain Tissue

In the hippocampal tissue obtained from autopsies, CBD displaced [^3^H]-8-OH-DPAT from its binding sites in a concentration-dependent manner (IC_50_ = 129.40 ± 9.40 μM; p*K*_i_ = 4.29 ± 0.03). In this tissue, the Hill coefficient was 3.37 ± 0.27. The radioligand displacement assay on the hippocampal tissue of patients with DR-MTLE revealed similar values (IC_50_ = 93.61 ± 18.25 μM, *p* = 0.1194; p*K*_i_ = 4.47 ± 0.09, *p* = 0.0748) as those observed in the autopsies. However, in the group of DR-MTLE patients, the Hill coefficient was higher (4.84 ± 0.35, *p* < 0.05) than in the group of autopsies.

**Figure 1:**
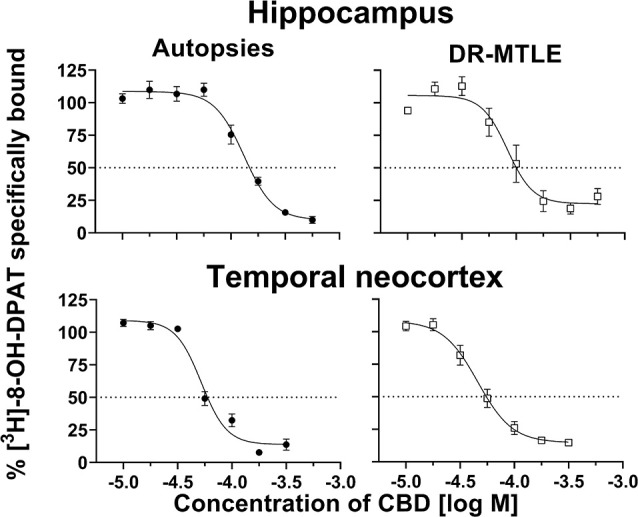
Effects of cannabidiol (CBD) on specific binding of [^3^H]-8-OH-DPAT to cell membranes obtained from the hippocampus (top panels) and temporal neocortex (bottom panels) of autopsies and patients with drug-resistant mesial temporal lobe epilepsy (MTLE; left and right panels, respectively). Symbols represent the mean ± Standard Error of the Mean (SEM) of five experiments. The dotted lines indicate 50% inhibition of specific binding. Curves were fitted to a model of four parameters (see “Materials and Methods” section).

In the temporal neocortex, we observed similar values in both DR-MTLE and autopsy groups (Figure 1). In the autopsy group, CBD displacement values (IC_50_ = 54.34 ± 3.66 μM; p*K*_i_ = 4.67 ± 0.03; Hill coefficient = 4.67 ± 0.93) were found within the same range of concentration as in the group of patients with DR-MTLE (IC_50_ = 46.84 ± 6.30 μM, *p* = 0.334; p*K*_i_ = 4.74 ± 0.05, *p* = 0.2643; Hill coefficient = 3.18 ± 0.57, *p* = 0.2091; Table 3).

**Table 3:** Data obtained from the radioligand displacement assay in the hippocampus and temporal neocortex of autopsies and patients with drug resistance mesial lobe epilepsy.

Brain structure	Group	IC_50_ (μM)	*K*_i_ (μM)	*pK*_i_	Hill coefficient
Hippocampus	Autopsies	129.40 ± 9.40	51.31 ± 3.73	4.29 ± 0.03	3.37 ± 0.27
	Patients	93.61 ± 18.25	37.12 ± 7.24	4.47 ± 0.09	4.84 ± 0.35*
Temporal neocortex	Autopsies	54.34 ± 3.66	21.55 ± 1.45	4.67 ± 0.03	4.67 ± 0.93
	Patients	46.84 ± 6.30	18.57 ± 2.50	4.74 ± 0.05	3.18 ± 0.57

IC_50_, inhibitory concentration 50; *K*_i_, inhibition constant; p*K*_i_, inverse logarithm of *K*_i_. *K*_i_ was determined from IC_50_ according to the Cheng–Prusoff equation (1973): *K*_i_ = IC_50_/(1 + [radioligand]/*K*_D_). Values are expressed as the mean ± SEM of five experiments. *p < 0.05 vs. autopsies.

### CBD Modifies the Activity of G_i/o_ Protein-Coupled Receptors

**Figure 2:**
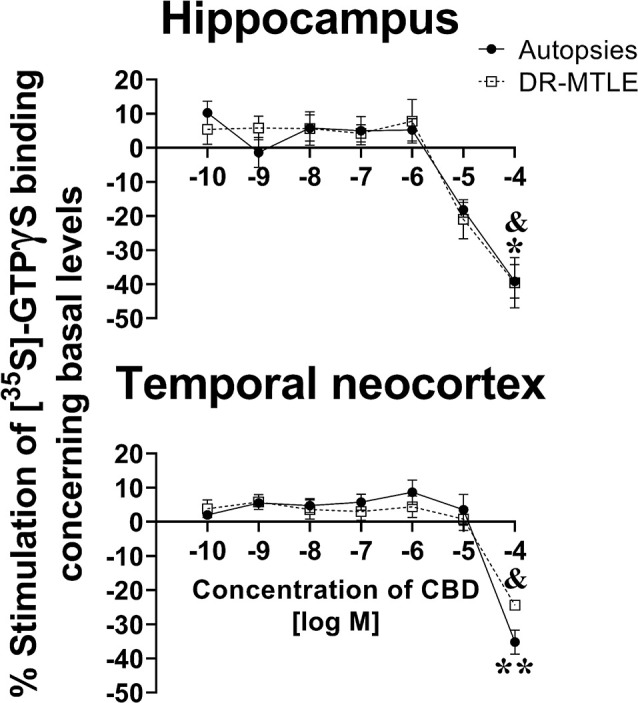
Effect of increasing concentrations of CBD on [^35^S]-GTPγS binding in cell membranes obtained from the hippocampus (top panels) and the temporal neocortex (bottom panels) of autopsies and patients with drug-resistant MTLE. Values are expressed as the mean ± SEM. *p < 0.05, **p < 0.01 (autopsies vs. baseline values); ^&^*p* < 0.05 MTLE vs. baseline values.

In the autopsies, CBD did not produce significant changes in the binding of [^35^S]-GTPγS at low concentrations (1 pM to 10 μM, *p* > 0.05), neither in the hippocampus nor in the temporal neocortex. However, at high concentrations (100 μM), CBD reduced [^35^S]-GTPγS binding, which was 39.2% and 35.2% lower in the hippocampus and temporal neocortex, respectively, than the basal levels (*p* < 0.05 and *p* < 0.01, respectively; Figure 2).

When evaluating the brain tissue of patients with DR-MTLE, the [^35^S]-GTPγS assay revealed similar results as those observed in the group of autopsies. No changes were detected at low concentrations (1 pM to 10 μM, *p* > 0.05), whereas 100 μM CBD induced a significant reduction of [^35^S]-GTPγS binding in the hippocampus and temporal neocortex (39.6% and 24.4% lower, respectively, when compared to basal binding levels, *p* < 0.05; Figure 2). In comparison with the autopsy group, a less evident [^35^S]-GTPγS binding decrease induced by CBD was observed in the temporal neocortex of the DR-MTLE group (*p* < 0.05).

**Figure 3:**
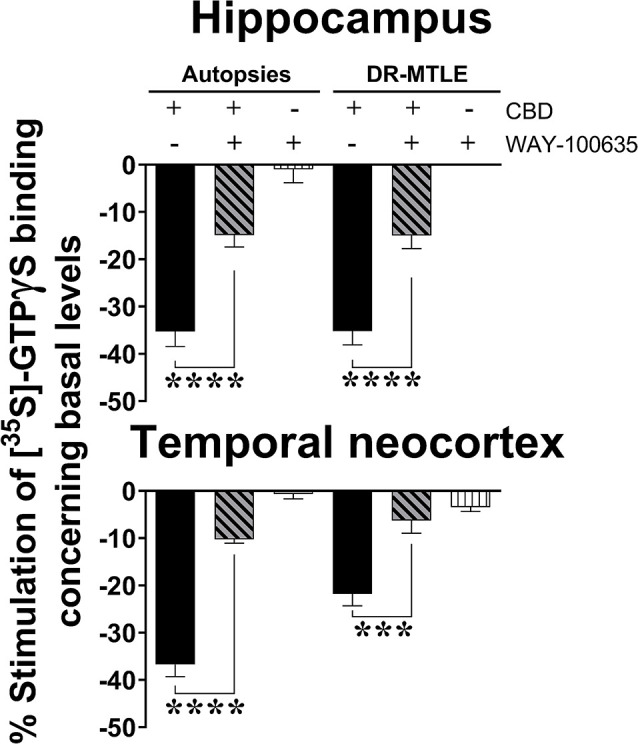
Effect of CBD (100 μM) on the constitutive activity of G_i/o_ proteins alone and combined with WAY100635 on [^35^S]-GTPγS binding in cell membranes obtained from the hippocampus (top panel) and temporal neocortex (bottom panel) of autopsies and patients with drug-resistant MTLE. Values are expressed as the mean ± SEM. ***p < 0.001; ****p < 0.0001.

The decrease in the constitutive activity of G_i/o_ proteins induced by a high concentration of CBD was partially reversed in the presence of WAY-100635 at 100 μM in both the autopsy group (hippocampus, 59.8%; *p* < 0.0001; temporal neocortex, 71.5%; *p* < 0.0001) and the DR-MTLE group (hippocampus, 53.7%; *p* < 0.0001; temporal neocortex, 68.5%; *p* < 0.001; Figure 3).

## Discussion

The radioligand displacement assay results support CBD affinity for 5-HT_1A_ receptors in the brain tissue obtained from autopsies and patients with DR-MTLE. Furthermore, the experiments using [^35^S]-GTPγS revealed that CBD decreased the constitutive activity of G_i/o_ protein-coupled receptors at high concentrations and an antagonist of 5-HT_1A_ receptors significantly reversed this effect.

Our experiments showed that CBD displaces [^3^H]-8-OH-DPAT from its binding site on 5-HT_1A_ receptors in a concentration-dependent manner in human hippocampal and neocortical samples. These findings support the affinity of CBD for 5-HT_1A_ receptors in the human brain. Indeed, they are in agreement with previous studies indicating that the exposure to CBD at micromolar concentrations displaced [^3^H]-8-OH-DPAT from its binding site in CHO cells expressing the human 5-HT_1A_ receptor (Russo et al., 2005).

The present results indicate that CBD interacts with 5-HT_1A_ receptors at high concentrations regardless of its lower affinity (p*K*_i_ ≈ 4.5) in comparison with other ligands such as serotonin (p*K*_i_ = 9.2), 8-OH-DPAT (p*K*_i_ = 8.0), or WAY-100635 (p*K*_i_ = 8.7; U.S. National Library of Medicine). However, the Hill coefficients obtained suggest that CBD is an allosteric modulator of 5-HT_1A_ receptors (Hill, 1910; Goutelle et al., 2008). This condition may facilitate the binding of endogenous ligands and agonists to 5-HT_1A_ receptors (May et al., 2007; Saleh et al., 2018). Indeed, the allosteric condition of CBD may represent a novel therapeutic strategy to influence the effects of 5-HT_1A_ receptors (Azam et al., 2020).

Radioligand binding assay revealed differences in IC_50_ of CBD between hippocampus and temporal neocortex. It also showed the inversion of the Hill coefficient profile between autopsies and patients when temporal neocortex and hippocampus were compared. The results obtained suggest that activation of 5-HT_1A_ receptors by CBD induces different functional consequences within these brain regions and depending on the pathological condition. It is known that variations of Hill coefficients are associated with conformational changes of the receptor and the number of binding sites (Colquhoun, 1998; Prinz, 2010). We found that the group of DR-MTLE patients showed a higher Hill coefficient in the hippocampus, the brain structure that mainly develops aberrant changes due to epilepsy (Bartolomei et al., 2008). This result indicates that epilepsy might be producing changes in the binding sites for CBD on the 5-HT_1A_ receptors in the hippocampus (Lolkema and Slotboom, 2015). This finding is consistent with a previous study in which the Hill coefficient increase was associated with neuronal hyperexcitability and seizure activity due to a conformational change in the γ2 subunit of the GABA_A_ receptor (Migita et al., 2013). Further binding kinetics studies are necessary to determine changes mediating the enhanced Hill coefficient in 5-HT_1A_ receptors in the hippocampus of patients with DR-MTLE.

Regarding the [^35^S]-GTPγS assay, low concentrations of CBD did not modify the activity of G_i/o_ proteins in cell membranes obtained from the human brain, neither in autopsies nor in patients with DR-MTLE. These results are similar to those previously reported by Rock et al. (2012), who described that G_i/o_ protein-coupled receptors in rat brainstem membranes were not activated at low concentrations of CBD. This finding could be explained because the receptor–transducer selectivity was not evaluated. This is an important limitation of the experimental procedure used in the present study since the [^35^S]-GTPγS assay evaluates all the interactions of G_i/o_ protein-coupled receptors. Another possibility is that, at low concentrations of CBD, an equilibrium state between the activation and inhibition of G_i/o_ proteins is achieved, which is a common condition for the G_i/o_ protein-coupled receptors (Seyedabadi et al., 2019).

The constitutive activity of 5-HT_1A_ receptors is susceptible to the effect of inverse agonists (Newman-Tancredi et al., 1997; Milligan, 2003). We found that high concentrations of CBD (100 μM) decreased the binding of [^35^S]-GTPγS to G_i/o_ proteins below the baseline values. Therefore, CBD reduced the constitutive activity of receptors coupled to G_i/o_ proteins. These changes were partially reversed when cell membranes were exposed to CBD in the presence of WAY100635, an antagonist of 5-HT_1A_ receptors. The results obtained indicated that at high concentrations, CBD modifies the constitutive activity of 5-HT_1A_ receptors acting as an inverse agonist.

Inverse agonists could indirectly increase the signalling of targeted receptors through the increase in the proportion of receptors on the cellular surface (Abbas et al., 2007; Kumar et al., 2019). Therefore, the continuous administration of inverse agonists of 5-HT_1A_ receptors induces antiallodynic effects due to inverse tolerance (Deseure et al., 2003). According to this information, the continuous administration of CBD as an inverse agonist of 5-HT_1A_ receptors may represent a therapeutic strategy to augment the signaling of these and other G_i/o_ protein-coupled receptors involved in neuroprotection. Additional experiments are essential to support this notion.

It is known that CBD is an inverse agonist of other G_i/o_ protein-coupled receptors, such as CB2 receptors (Thomas et al., 2007) and GPR3 and GPR6 orphan receptors (Laun and Song, 2017). In the present study, WAY-100635 partially blocked the CBD-induced decreased binding of [^35^S]-GTPγS to G_i/o_ proteins. WAY-100635 is considered the quintessential antagonist of the 5-HT_1A_ receptors (*K*_i_ = 2.2 nM). However, although with lower affinity values, it is also an antagonist of other receptors such as 5-HT_2A_ (*K*_i_ = 6260 nM), 5-HT_2B_ (*K*_i_ = 24 nM), and D2-like (*K*_i_ 16.4–940 nM; Chemel et al., 2006). Therefore, WAY-100635 could be blocking the constitutive activity of 5-HT_1A_, 5-HT_2A_, 5-HT_2B_, and D2-like receptors as well. On the other hand, [^3^H]-8-OH-DPAT has low affinity for 5-HT_7_ (Thomas et al., 1998) and α1 receptors (Yoshio et al., 2001). Then, some of the results obtained can involve the action of CBD on these receptors. Future experiments should be conducted to investigate the effects of CBD on binding and constitutive activity of different type of receptors in the brain of patients with epilepsy. These experiments should include kinetic binding studies and displacement experiments (pseudo-competition experiments) in the presence and absence of CBD.

5-HT_1A_ receptors play an important role in cerebral functions, and they are considered targets to develop novel therapeutic strategies. They show heterogeneous distribution, including pre- and postsynaptic localization, as well as cross-talk with different types of 5-HT and other neurotransmitter receptors. Dysfunction of 5-HT_1A_ receptors is associated with psychiatric disorders such as anxiety and depression (Popova and Naumenko, 2013). Experimental evidence indicates decline of 5-HT_1A_ receptor binding in the brain of patients with epilepsy (Toczek et al., 2003; Theodore et al., 2012). It is suggested that 5-HT_1A_ receptors on astrocytes represent a potential therapeutic target for the treatment of neurodegenerative disorders (Miyazaki and Asanuma, 2016). An important limitation of the present study is that the current methodology does not allow one to identify in which type of cells the 5-HT_1A_ receptors were evaluated. More experiments are needed to investigate the effects of CBD on specific brain cells and its therapeutic relevance.

## Data Availability Statement

The raw data supporting the conclusions of this article will be made available by the authors, without undue reservation.

## Ethics Statement

The studies involving human participants were reviewed and approved by Ethics committee of the Hospital General de Mexico and Centro de Investigacion y de Estudios Avanzados. The patients/participants provided their written informed consent to participate in this study.

## Author Contributions

LR conceived and designed the study and wrote the manuscript. CM-A carried out the experiments and analyzed the results. FC-C carried out the experiments. FV performed the neurosurgery of patients. AV and GA-C identified and evaluated the patients with epilepsy. MC-H collected samples, participated in the analysis of the results, and wrote the manuscript. All authors contributed to manuscript revision, read, and approved the submitted version.

## Conflict of Interest

The authors declare that the research was conducted in the absence of any commercial or financial relationships that could be construed as a potential conflict of interest.
